# Lung Auscultation for Detecting Interstitial Lung Disease in Patients with Newly Diagnosed Systemic Sclerosis: Retrospective Cohort Study

**DOI:** 10.3390/diagnostics16111577

**Published:** 2026-05-22

**Authors:** Felix W. Wireko, Vasilios Tzilas, Comfort Anim-Koranteng, Ahmed S. Sayed Ahmed, Yvette A. Yeboah-Kordieh, Ashima Makol, Jay H. Ryu

**Affiliations:** 1 Division of Pulmonary and Critical Care Medicine, Mayo Clinic, 200 First St. SW, Rochester, MN 55905, USA; 2 2nd Pulmonary Medicine Department, General University Hospital “Attikon”, Athens Medical School, National and Kapodistrian University of Athens, Rimini 1, 124 62 Athens, Greece; tzilasvasilios@gmail.com; 3 Division of Rheumatology, Allergy and Immunology, University of Cincinnati Medical Center, 3230 Eden Avenue, Cincinnati, OH 45267, USA; 4 Department of Medicine, MedStar Union Memorial Hospital, 201 E University Parkway, Baltimore, MD 21218, USA; 5 Division of Pulmonary, Critical Care and Sleep, Icahn School of Medicine at Mount Sinai, 1 Gustave L. Levy Place, New York, NY 10029-5674, USA; 6 Division of Rheumatology, Mayo Clinic, 200 First St. SW, Rochester, MN 55905, USA

**Keywords:** crackles, interstitial lung disease, lung auscultation, pulmonary function, systemic sclerosis

## Abstract

**Background/Objectives:** Interstitial lung disease (ILD) occurs commonly in systemic sclerosis (SSc) and is the leading cause of mortality. There are limited data on the accuracy of lung auscultation in identifying the presence of ILD in patients with SSc. **Methods:** We retrospectively identified patients with newly diagnosed SSc who had documented lung auscultation findings and chest high-resolution computed tomography (HRCT) available for review. Diagnoses were made by rheumatologists at Mayo Clinic, Rochester, Minnesota, USA over a 4-year period. Pulmonary function measurements included lung volumes, spirometry, and single-breath diffusing capacity. **Results:** Among 151 patients with SSc (median age, 62 years), 72.2% were female and 55.0% were never smokers. Limited cutaneous SSc was the most common phenotype (67.3%). Seventy (46.4%) patients were evaluated by pulmonologists. There was evidence of ILD by HRCT in 69 patients (45.7%); the most common pattern of ILD was fibrotic nonspecific interstitial pneumonia (59.2%). Respiratory symptoms were present in 46.4% of those with ILD compared to 15.9% among those without. The sensitivity and specificity for crackles heard by rheumatologists in detecting ILD were 50.7% and 97.6%, respectively; for pulmonologists, 71.4% and 85.7%, respectively. Presence of crackles was associated with high positive predictive values (94.6% for rheumatologists vs. 92.1% for pulmonologists, respectively); negative predictive values were moderate (70.2% vs. 56.3%, respectively). Crackles correlated with lower pulmonary function measures but did not differ across ILD patterns. **Conclusions:** Detection of crackles on lung auscultation appears to be a specific and moderately sensitive indicator of ILD (often asymptomatic) in patients with newly diagnosed SSc. The presence of crackles correlates with worse pulmonary function but may be absent in early ILD.

## 1. Introduction

Systemic sclerosis (SSc) is a complex, chronic, multisystem disease characterized by immune dysregulation, vasculopathy, and progressive fibrosis with excessive collagen deposition involving the skin and internal organs [1]. The disease is primarily categorized as limited cutaneous systemic sclerosis (lcSSc) or diffuse cutaneous systemic sclerosis (dcSSc) based on the extent of skin involvement. There is acral distribution in limited form, and involvement of the trunk and proximal limbs in diffuse form [2]. Systemic sclerosis sine scleroderma (ssSSc) is a variant that applies to individuals with typical SSc features (positive autoantibodies, Raynaud’s phenomenon, lung involvement) without skin manifestations [2].

Interstitial lung disease (ILD) occurs frequently in SSc (SSc-ILD) and is seen in about 35% to 52% of patients [3]. It is the leading cause of SSc-related mortality, accounting for 20% to 40% of deaths [4]. SSc-ILD carries a mortality risk almost three times higher than for those without ILD [5]. Risk factors for SSc-ILD include African American ethnicity, male sex, older age at disease onset, presence of anti-Scl-70/anti-topoisomerase I antibody, absence of anticentromere antibody, and dcSSc [1,6]. Early detection of ILD in SSc patients can be difficult, considering most patients have no respiratory symptoms in the earlier stages. Current expert consensus recommends screening all SSc patients for ILD through clinical assessment, pulmonary function testing (PFT), and chest high-resolution computed tomography (HRCT) [4,7]. Although lung function measures eventually become abnormal as ILD progresses, PFT lacks the sensitivity and specificity for diagnosing early ILD in patients with SSc [8]. The role of lung auscultation as a screening tool for ILD during clinical evaluation of patients with SSc is unclear.

Fine inspiratory crackles are commonly heard on physical examination of patients with fibrotic ILDs [9]. Lung auscultation is a readily available screening tool for ILD, particularly in low-resource settings, and would avoid costs and exposure to ionizing radiation associated with CT scanning. In the absence of available data on the accuracy of lung auscultation in screening for ILD in patients with SSc, we sought to address this issue by retrospectively evaluating patients with newly diagnosed SSc encountered in clinical practice. Thus, the aim of this study was to assess the utility of lung auscultation for detecting ILD patients newly diagnosed with SSc in a real-world setting.

## 2. Patients and Methods

This research study was approved by the Mayo Clinic Institutional Review Board (IRB) and deemed exempt from the requirement for IRB approval, with the need for written informed consent waived (IRB number 23-009787, 24 October 2023) since only existing data from medical records were used without patient contact.

**Figure 1:**
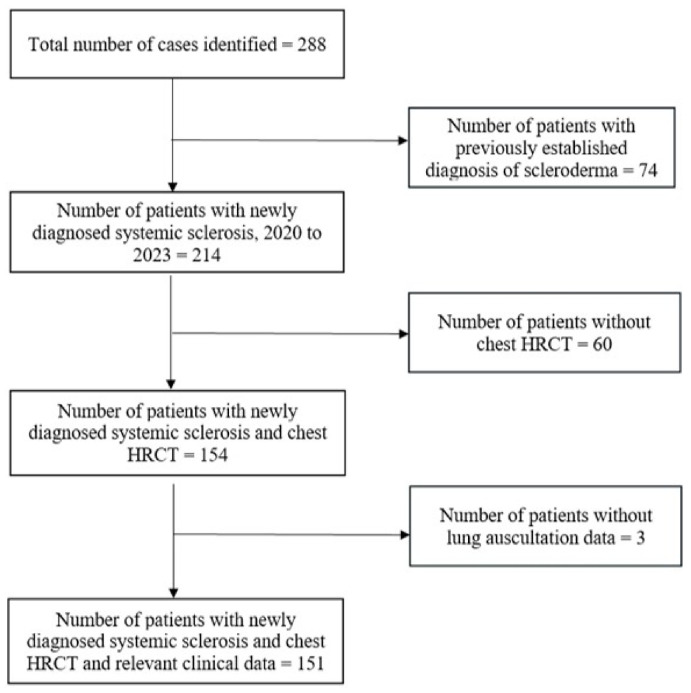
Patient cohort selection diagram. Of 288 patients identified, 214 had newly diagnosed systemic sclerosis between 2020 and 2023. After excluding those without chest HRCT or relevant clinical data, 151 patients were included in the final analysis.

This retrospective cohort study incorporated all patients newly diagnosed with SSc by rheumatologists at Mayo Clinic in Rochester, Minnesota, during a 4-year period between January 2020 and December 2023, with chest HRCT study from initial diagnosis available for current review. We identified 288 subjects; 74 had been diagnosed with scleroderma previously and were excluded (Figure 1).

Sixty-three additional subjects were excluded for reasons shown in Figure 1. Thus, the final cohort comprised 151 subjects with newly diagnosed SSc with chest HRCT available for current review and lung auscultatory findings documented in the medical records.

Demographic data (age, sex, race, smoking status), clinical features (SSc phenotype, comorbidities, respiratory symptoms, presence of inspiratory crackles (auscultated by rheumatologist and/or pulmonologist and other physical findings, body mass index), laboratory data (autoimmune serologies including antinuclear antibody [ANA], anti-topoisomerase 1, anti-centromere, anti-RNP and anti-RNA polymerase III), and PFT results were retrieved from electronic medical records. Clinical evaluation including lung auscultation occurred in a quiet examination room during their consultation visit in the outpatient clinic. Clinicians used stethoscopes of their personal preference. Referral to pulmonology was made at the discretion of the evaluating rheumatologist and patient preference most commonly.

Chest CT scans were performed on available clinical systems including Siemens SOMATOM Force 192-slice dual-source scanner, Siemens Definition Flash 128-slice dual-source scanner, and Siemens NAEOTOM Alpha 288-slice photon-counting dual-source scanner (Siemens Healthineers, Forchheim, Germany) using thin-section (≤1.5 mm) reconstructions and high-spatial frequency reconstruction algorithm. HRCT scans of the chest were reviewed independently by two of the authors (VT, JHR) to confirm the presence or absence of ILD as indicated in the original chest radiologist’s interpretation, and to categorize the pattern of ILD as usual interstitial pneumonia (UIP), probable UIP, fibrotic nonspecific interstitial pneumonia (NSIP), cellular NSIP, organizing pneumonia, pleuroparenchymal fibroelastosis, and mild nonspecific ILD (Table 1).

Discrepancies in interpretations of HRCT findings (N = 11, 7.3%) were reconciled by consensus on second review conducted by two of the authors (VT, JHR).

Pulmonary function measurements (lung volumes, spirometry, and single-breath diffusing capacity) were obtained using the Jaeger SentrySuite™ Body Plethysmograph system (Jaeger Medical, Basingstoke, UK). Analysis of PFT data included forced vital capacity (FVC), diffusing capacity of the lungs for carbon monoxide (DLCO), and total lung capacity (TLC). TLC percent (%) predicted was categorized as normal (≥80), mild (70–79), moderate (50–69), and severe (<50). Similarly, percent predicted DLCO was classified as normal (>75), mild (60–75), moderate (40–59), and severe (<40). FVC percent predicted was categorized as normal (≥80) or abnormal (<80), with reference values from the National Health and Nutrition Examination Survey III.

### Statistical Analysis

The baseline characteristics of the study participants were summarized based on the presence or absence of ILD on chest HRCT. Continuous variables are presented as mean (±standard deviation [SD]) or median (range), while categorical variables are expressed as proportions. Student’s t-test and Wilcoxon signed-rank test were employed for normally and non-normally distributed continuous variables, respectively. Chi-square tests were utilized for categorical variables. The lung auscultation findings were analyzed, and the association between inspiratory crackles and SSc-ILD was examined using a multivariable analysis (Poisson models). Statistical analyses were conducted using STATA 17 (Stata Corp, Grand Forks, ND, USA), with a two-sided *p*-value of 0.05, to determine the significance of the results.

## 3. Results

The study cohort of 151 patients comprised 109 (72.2%) females, 132 (88.1%) whites, and 83 (55.0%) never smokers, with a median (range) age at diagnosis of 62 (22–88) years (Table 2). Limited SSc was the most common SSc subtype, 101 (67.3%). Forty-five patients (29.8%) had respiratory symptoms (cough and/or dyspnea).

HRCT of the chest demonstrated evidence of ILD in 69 (45.7%) patients, among whom the most prevalent ILD pattern was nonspecific interstitial pneumonia (NSIP), seen in 49 (71.0%) of these patients and predominantly the fibrotic-NSIP (f-NSIP) subtype, seen in 41 (59.4%).

ANA positivity was documented in nearly all patients, with and without evidence of ILD on HRCT. High anti-topoisomerase 1 (Scl-70) antibody level (>8 U) was significantly more common in those with ILD compared to those without ILD, 20.9% versus 1.2%, respectively (*p*-value < 0.001). High level of anticentromere antibodies (>8 U) was less frequent in those with ILD compared to those without ILD, 17.6% versus 37.7%, respectively (*p* value = 0.014).

Inspiratory crackles on lung auscultation were documented by rheumatologists in 37 of 151 (24.5%) patients, and 35 (94.6%) of this cohort had HRCT evidence of ILD (Table 3). Among 114 patients in whom the rheumatologists heard no inspiratory crackles, 34 (29.8%) patients had CT evidence of ILD. Sensitivity and specificity of inspiratory crackles heard by rheumatologists in detecting ILD were 50.7% and 97.6%, respectively.

Seventy (46.4%) of 151 patients had been evaluated by pulmonologists; inspiratory crackles were documented in 38 (54.3%) patients and 35 (92.1%) of the 38 had CT evidence of ILD (Table 4). Among 32 subjects in whom no inspiratory crackles were heard by the pulmonologists, 14 (43.8%) patients had CT evidence of ILD. Sensitivity and specificity of inspiratory crackles heard by pulmonologists in detecting ILD were 71.4% and 85.7%, respectively.

The positive predictive values (PPV) for crackles heard by rheumatologists and pulmonologists were high (94.6% vs. 92.1%, respectively). Conversely, the negative predictive values for absence of crackles were moderate (70.2% vs. 56.3%, respectively).

Respiratory symptoms (cough and/or dyspnea) were present in 46.4% of those with ILD compared to 15.9% among those without ILD. However, 53.6% of patients with ILD had neither dyspnea nor cough. Among the patients with ILD, dyspnea was documented in 30 (43.5%) and 16 (23.2%) with cough. Among those without ILD, dyspnea was documented in 11 (13.4%) and cough in 6 (7.3%). Both dyspnea (*p* < 0.001) and cough (*p* < 0.012) showed a significant correlation with the presence of ILD, with dyspnea showing a stronger association.

As expected, the mean % predicted of TLC, DLCO, and FVC were significantly lower in patients with ILD compared to those without ILD, 77.0 vs. 95.0 (95% CI: 13.20 to 22.80, *p* < 0.001), 63.0 vs. 82.0, (95% CI: 14.20 to 23.80, *p* <0.001), and 79.4 vs. 94.0 (95% CI: 10.60 to 20.20, *p* < 0.001), respectively (Table 3 and Table 4).

Among 69 patients with ILD, those who had crackles heard by rheumatologists manifested a lower mean % predicted TLC (*p* = 0.004) and DLCO (*p* = 0.025) compared to those with no crackles heard on lung auscultation. In the subset of 49 patients with ILD evaluated by pulmonologists, those who manifested crackles showed a trend toward lower mean % predicted TLC (*p* < 0.056) and DLCO (*p* < 0.08) compared to those without crackles, but no difference in % predicted FVC (*p* < 0.589).

Among patients with ILD, there was no correlation between the ILD patterns seen on HRCT and crackles heard by rheumatologists (*p* = 0.168) or pulmonologists (*p* = 0.138). The Spearman correlation between ILD pattern and DLCO severity was approximately −0.317 (*p* = 0.015), and between ILD pattern and TLC was −0.391 (*p* = 0.005). The negative correlations suggest that as the ILD pattern progresses from cellular NSIP (c-NSIP) to fibrotic NSIP (f-NSIP) to probable UIP, both DLCO and TLC tend to decrease. This reflects worsening lung function, with more advanced fibrotic patterns associated with greater impairment in gas exchange and lung volume.

Multivariable regression analysis assessed the relationship between ILD present on HRCT scans and crackles heard by rheumatologists and pulmonologists on lung auscultation (Table 5 and Table 6). In this multivariable analysis adjusted for age, sex, body mass index, spirometry, and anti-topoisomerase 1 antibody levels, a significant correlation was observed between ILD on HRCT and crackles identified by rheumatologists (IRR 1.643, 95% CI 1.069–2.526, *p* = 0.024), while crackles detected by pulmonologists showed a trend toward significance (IRR 1.624, 95% CI 0.969–2.721, *p* = 0.066). Other clinical variables, pulmonary function measures and anti-topoisomerase levels did not appear to correlate with the presence of ILD on HRCT.

## 4. Discussion

In this retrospective study, we examined the accuracy of lung auscultation in detecting the presence of ILD in 151 patients with newly diagnosed SSc in real-world clinical practice, nearly one-half of whom had HRCT evidence of ILD, often in the absence of respiratory symptoms. Our study demonstrated presence of inspiratory crackles to have a moderate sensitivity and a high specificity in detecting ILD in these patients. The presence of crackles tended to correlate with lower pulmonary function measures.

In a prospective study of 290 patients referred to an ILD clinic in Canada, crackles (“fine” and/or “coarse”) were documented by clinicians in 98% patients with idiopathic pulmonary fibrosis (IPF) and 84% of those with non-IPF ILD [10]. In a study of patients with fibrotic ILDs, “Velcro-type” crackles correlated with the presence of fibrotic ILD, particularly the UIP pattern of ILD [9]. In addition, lung auscultation has been identified as a useful noninvasive method for screening industrial populations exposed to asbestos [11]. Among patients with SSc and evidence of ILD by HRCT, less than half were reported to have a positive clinical examination (“presence of basilar Velcro-like crackles”) [12]. Thus, the prevalence of crackles appears to vary depending on the underlying ILD and clinical setting. In our retrospective study cohort of patients with newly diagnosed SSc encountered in real-world practice, quality of crackles was often not characterized as “fine” or “Velcro-like” versus “coarse.” Thus, we were not able to specifically address the value of Velcro-like” crackles.

Among 69 patients who had SSc-ILD in our study, no crackles were heard by rheumatologists in nearly one-half of patients. Among 49 patients with SSc-ILD evaluated by pulmonologists, no crackles were heard in over one-quarter of patients. With the advent of advanced chest imaging, particularly HRCT, there has been lessening emphasis on lung auscultation and perhaps a decline in auscultatory skills. For example, fine inspiratory (‘Velcro”) crackles may be difficult to appreciate by the busy clinician unless the patient is deliberately instructed to take slow and deep breaths. Accordingly, the absence of crackles on lung auscultation does not exclude the presence of ILD.

Inspiratory crackles are commonly encountered in patients with fibrotic ILDs and have been reported to be more common than respiratory symptoms (dyspnea or cough) or abnormal PFT results (restrictive pattern or low diffusing capacity) [10]. However, inspiratory crackles are not specific for fibrotic processes in the lung and can be associated with other lung diseases including infections, noninfectious inflammation, edema, and others [13]. Nonetheless, in our cohort of newly diagnosed SSc patients, detection of inspiratory crackles appeared to be relatively specific for ILD. While the most common pattern of ILD seen on HRCT was fibrotic NSIP, specific patterns of ILD did not correlate with the presence of crackles.

In recent years, the digital stethoscope has been developed which may facilitate respiratory sound analysis with the aid of artificial intelligence techniques [14]. This type of auscultatory analysis would reduce subjectivity and interobserver variability and is a promising avenue for more accurate clinical assessment. In a recent systematic review of screening methods used in detecting ILD in patients with rheumatoid arthritis-associated ILD, digital auscultation achieved diagnostic accuracy of 84% to 90% compared to HRCT, with a sensitivity of 93% and specificity of 77% to 88% [15]. The sensitivity and specificity of PFT variables (DLCO, TLC, and FVC) were 0.77 and 0.70, respectively, in our study and were comparable to values previously reported in the literature, where a PFT-based ILD prediction tool showed a sensitivity of 0.79 and specificity of 0.83 [16]. A recent review on lung sound analysis describes the potential role of AI-based auscultation for early and accurate diagnosis of ILD [17].

The limitations of our study relate to the research setting and retrospective design. This study was conducted in a tertiary referral medical center and might not reflect the general scleroderma population. Lung auscultation data were retrieved from medical records as physicians documented them. The crackles were often not categorized as fine or coarse. Our patient population was predominantly white (88.1%), which may limit applicability to other racial groups.

## 5. Conclusions

Detection of inspiratory crackles on lung auscultation in patients with newly diagnosed SSc strongly indicates the presence of ILD even in the absence of respiratory symptoms. The presence of crackles correlated with worse pulmonary function measures, including lower TLC and DLCO. However, crackles were not associated with specific ILD patterns and may be absent in early disease.

## Figures and Tables

**Table 1:** Defining criteria for interstitial lung disease patterns on HRCT chest.

ILD Pattern	Radiologic Features
UIP	Reticular pattern with traction bronchiectasis/bronchiolectasis Honeycombing Basal, subpleural predominant distribution Absence of features to suggest an alternative diagnosis
Probable UIP	Reticular pattern with traction bronchiectasis/bronchiolectasis No honeycombing Basal, subpleural predominant distribution Absence of features to suggest an alternative diagnosis
Fibrotic NSIP	Bilateral reticular opacities and/or GGOs with traction bronchiectasis/bronchiolectasis No honeycombing ±subpleural sparing
Cellular NSIP	Bilateral GGOs without traction bronchiectasis/bronchiolectasis ±subpleural sparing
Organizing Pneumonia	Patchy consolidative opacities ±GGOs
Pleuroparenchymal fibroelastosis	Subpleural consolidative opacities with traction bronchiectasis in the upper lobes with architectural distortion, upper lobe volume loss
Mild nonspecific ILD	Mild reticular and/or GGOs without traction bronchiectasis/bronchiolectasis No honeycombing Basal, subpleural predominant distribution

GGOs = ground-glass opacities.

**Table 2:** Demographics, scleroderma types and autoimmune workup (N = 151).

	Total N (%)	ILD 69 (45.7)	No ILD 82 (54.3)	*p* Value
Age at diagnosis, median (range), years	62 (22–88)	64 (22–88)	58.5 (29–83)	0.060
Female	109 (72.2)	43 (62.3)	66 (80.5)	0.018
Race				0.414
White	133 (88.1)	61 (88.4)	72 (87.8)	
Black/African American	8 (5.3)	2 (2.9)	6 (7.3)	
Other	10 (6.6)	6 (8.7)	4 (4.9)	
Smoking status				0.05
Current	10 (6.6)	1 (1.4)	9 (11.0)	
Former	58 (38.4)	30 (43.5)	28 (34.1)	
Never	83 (55.0)	38 (55.1)	45 (54.9)	
BMI	26.1	26.2	26.0	0.988
Symptoms				
Dyspnea	41 (27.2)	30 (43.5)	11 (13.4)	<0.001
Cough	22 (14.6)	16 (23.2)	6 (7.3)	<0.02
Neither	106 (70.2)	37 (53.6)	69 (84.1)	
Scleroderma pattern				0.805
Diffuse scleroderma	31 (20.5)	15 (21.7)	16 (19.5)	
Limited scleroderma	102 (67.5)	47 (68.1)	55 (67.1)	
Sine scleroderma	18 (12.0)	7 (10.2)	11 (13.4)	
ILD pattern				
f-NSIP		41 (59.4)		
c-NSIP		8 (11.6)		
Mild nonspecific ILD		17 (24.6)		
PPFE		1 (1.5)		
Probable UIP		1 (1.5)		
UIP		1 (1.5)		
ANA				
Positive	144 (94.7)	64 (94.8)	80 (97.5)	0.162
ANA pattern				<0.001
Centromere	53 (41.1)	15 (27.3)	38 (51.3)	
Homogenous	21 (16.3)	18 (32.7)	3 (4.1)	
Nucleolar	11 (8.5)	2 (3.6)	9 (12.2)	
Speckled	44 (34.1)	20 (36.4)	24 (32.4)	
Anti topoisomerase 1 antibody, U				<0.001
<0.2	123 (83.7)	49 (73.1)	74 (92.5)	
0.2–8	9 (6.1)	4 (6.0)	5 (6.2)	
>8	15 (10.2)	14 (20.9)	1 (1.3)	
Anticentromere antibody, U				0.014
<0.2	100 (68.9)	55 (80.9)	45 (58.4)	
0.2–8	4 (2.8)	1 (1.5)	3 (3.9)	
>8	41 (28.3)	12 (17.6)	29 (37.7)	
RNA polymerase antibody, U				0.265
<10	102 (69.9)	45 (68.2)	57 (71.3)	
10–50	17 (11.6)	10 (15.2)	7 (8.7)	
50–100	13 (8.9)	7 (10.6)	6 (7.5)	
100–150	8 (5.5)	1 (1.5)	7 (8.7)	
>150	6 (4.1)	3 (4.5)	3 (3.8)	

Data are presented as no. (%), unless otherwise indicated. ANA = antinuclear antibody; BMI = body mass index; c-NSIP = cellular nonspecific interstitial pneumonia; f-NSIP = fibrotic nonspecific interstitial pneumonia; ILD = interstitial lung disease; PPFE = pleuroparenchymal fibroelastosis; UIP = usual interstitial pneumonia.

**Table 3:** Correlation of crackles heard by rheumatologists, presence or absence of ILD, and pulmonary function values (N = 151).

	Total N (%)	ILD on CT 69 (45.7%)	No ILD on CT 82 (54.3%)	*p* Value
Crackles heard by rheumatologist, n (%)				<0.001
Yes	37 (24.5)	35 (50.7)	2 (2.4)	
No	114 (75.5)	34 (49.3)	80 (97.6)	
TLC % predicted, mean	86.0	77.0	95.0	<0.001
DLCO % predicted, mean	73.0	63.0	82	<0.001
FVC % predicted, mean	87.4	79.4	94.8	<0.001
FVC % predicted, n (%)				<0.001
<80	41 (32.8)	30 (50.0)	11 (16.9)	
≥80	84 (67.2)	0 (50.0)	54 (83.1)	

DLCO = diffusing capacity of the lung for carbon monoxide; FVC = forced vital capacity; ILD = interstitial lung disease; TLC = total lung capacity.

**Table 4:** Correlation of crackles heard by pulmonologists, presence or absence of ILD, and pulmonary function values (N = 70).

	Total N (%)	ILD on CT 49 (70.0%)	No ILD on CT 21 (30.0%)	*p* Value
Crackles heard by pulmonologist, n (%)				<0.001
Yes	38 (54.3)	35 (71.4)	3 (14.3)	
No	32 (45.7)	14 (28.6)	18 (85.7)	
TLC % predicted, mean	79	73.0	96.0	<0.001
DLCO % predicted, mean	64	59.0	75	<0.001
FVC % predicted, mean	80.1	75.0	93.5	<0.001
FVC % predicted, n (%)				<0.011
<80	29 (32.8)	26 (50.0)	3 (16.9)	
≥80	33 (67.2)	19 (50.0)	14 (83.1)	

DLCO = diffusing capacity of the lung for carbon monoxide; FVC = forced vital capacity; ILD = interstitial lung disease; TLC = total lung capacity.

**Table 5:** Correlation between ILD on CT and crackles heard by rheumatologists (N = 151).

	Adjusted IRR	95% CI	*p* Value
Crackles	1.643	1.069 to 2.526	0.024
Age at diagnosis	1.003	0.989 to 1.018	0.682
Sex	1.186	0.814 to 1.728	0.374
BMI	0.010	0/979 to 1.043	0.523
DLCO	0.996	0.982 to 1.010	0.598
FVC ≥ 80	0.871	0.422 to 1.797	0.709
TLC	0.986	0.960 to 1.013	0.308
Anti topoisomerase 1 (Scl 70), U			
0.2 to 8	0.497	0.124 to 1.986	0.323
>8	1.506	0.960 to 2.276	0.052

BMI = body mass index; CI = Confidence Interval; DLCO = diffusing capacity of lungs for carbon monoxide; FVC = forced vital capacity; ILD = interstitial lung disease; IRR = incidence rate ratio; TLC = total lung capacity.

**Table 6:** Correlation between ILD on CT and crackles heard by pulmonologists (N = 70).

	Adjusted IRR	95% CI	*p* Value
Crackles	1.624	0.969 to 2.721	0.066
Age at diagnosis	0.997	0.985 to 1.009	0.638
Sex	0.976	0.724 to 1.316	0.871
BMI	0.993	0.963 to 1.024	0.654
DLCO	0.999	0.982 to 1.018	0.989
FVC ≥ 80	0.854	0.453 to 1.610	0.625
TLC	0.987	0.960 to 1.015	0.371
Anti topoisomerase			
0.2 to 8	0.332	0.093 to 1.189	0.090
>8	1.029	0.743 to 1.424	0.865

BMI = body mass index; CI = Confidence Interval; DLCO = diffusing capacity of lungs for carbon monoxide; FVC = forced vital capacity; ILD = interstitial lung disease; IRR = incidence rate ratio; TLC = total lung capacity.

## Data Availability

All data generated or analyzed during this study are included in this article. Additional deidentified data are available from the corresponding author upon reasonable request.

## Abbreviations

The following abbreviations are used in this manuscript.

ANA	antinuclear antibody
DLCO	diffusing capacity of the lungs for carbon monoxide
dcSSc.	diffuse cutaneous systemic sclerosis
FVC	forced vital capacity
HRCT	high-resolution computed tomography
ILD	interstitial lung disease
lcSSc	limited cutaneous systemic sclerosis
PFT	pulmonary function testing
SD	standard deviation
ssSSc	systemic sclerosis sine scleroderma
SSc	systemic sclerosis
SSc-ILD	systemic sclerosis-associated interstitial lung disease
TLC	total lung capacity
